# Factors influencing warm ischemia time in robot-assisted partial nephrectomy change depending on the surgeon’s experience

**DOI:** 10.1186/s12957-022-02669-0

**Published:** 2022-06-15

**Authors:** Kazuyuki Numakura, Mizuki Kobayashi, Atsushi Koizumi, Soki Kashima, Ryohei Yamamoto, Taketoshi Nara, Mitsuru Saito, Shintaro Narita, Takamitsu Inoue, Tomonori Habuchi

**Affiliations:** 1 Department of Urology, Akita University Graduate School of Medicine, 1-1-1 Hondo, Akita, 010-8543 Japan; 2 Department of Urology, International University of Health and Welfare, Narita Hospital, 852 Hatakeda, Narita, 286-0124 Japan

**Keywords:** RENAL score, Kidney, Oncology, Robotic surgery

## Abstract

**Introduction:**

Warm ischemia time (WIT) is a primary concern for robot-assisted laparoscopic partial nephrectomy (RALPN) patients because longer WIT is significantly associated with postoperative deteriorating kidney function. Tumor complexity, determined by the RENAL nephrometry score (RENAL score), can help predict surgical outcomes, but it is unclear what RENAL score and clinical factors affect WIT. This study explored the clinical factors predicting long WIT in experienced surgeon to RALPN.

**Materials and methods:**

In our institute, 174 RALPNs were performed between November 2013 and February 2021, of which 114 were performed by a single surgeon and included in this study. Clinical staging and the total RENAL score were determined based on preoperative CT scans. The cases were divided into three groups based on experience: period 1: 1–38, period 2: 39–76, and period 3: 77–114. The clinical factors associated with longer WIT were analyzed per period.

**Results:**

The overall median tumor diameter was 32 mm, and one patient had a positive surgical margin, but there were no cancer-related deaths. In total, there were 18 complications (15.8%). Periods 2 and 3 had larger tumor diameters (*p* < 0.01) and worse preoperative kidney function (*p* = 0.029) than period 1. A RENAL L-component score of 3 was associated with longer WIT in period 3 (odds ratio: 3.900; 95% confidence interval: 1.004–15.276; *p* = 0.044), but the tumor diameter and the total RENAL score were not.

**Conclusions:**

A large tumor in the central lesion indicated by the RENAL L-component score was associated with increased WIT in RALPN.

**Supplementary Information:**

The online version contains supplementary material available at 10.1186/s12957-022-02669-0.

## Introduction

 After the introduction of the robotic platform for kidney surgery, robot-assisted laparoscopic partial nephrectomy (RALPN) for T1 renal cell carcinoma (RCC) became a standard procedure [1, 2]. There are obvious advantages to robotic surgery over pure laparoscopic nephrectomies, such as more precise resections and proper parenchyma suturing [3]. The robotic procedure also achieves good oncological outcomes, preserves more kidney function, and avoids complications [4].

A trifecta of RALPN was defined as warm ischemia time (WIT) ≤ 25 min, negative surgical margins, and no postoperative complications ≥ grade 2. This is a useful evaluation method for assuring good clinical outcomes in RALPN patients [5]. Of the three factors, WIT should be the most concerning because positive surgical margins and severe complications are quite rare. Moreover, a longer WIT is a major factor associated with deteriorating kidney function [6] and potentially leading to worse survival rates due to chronic kidney disease and cardiovascular disease [7].

The RENAL nephrometry score (radius [tumor size], exophytic/endophytic properties, nearness of the tumor to the collecting system, anterior/posterior, location relative to the polar line; RENAL score) is used to predict surgical difficulty in RALPN and laparoscopic partial nephrectomy patients [8]. However, it remains unclear what RENAL score or other clinical factors affect WIT in RALPN [9, 10]. Since most previous studies assessed a predicting factor of WIT only depending on dividing patients into groups by clinical factors and/or surgical techniques, a real factor could be covered up due to many confounders [11–13]. To minimize this influence, we assessed WIT by dividing patients into three chronological periods, which could reflect a learning curve [14], in the same surgical procedure by a single surgeon. This study aimed to delineate clinical factors predicting longer WIT during RALPN.

## Materials and methods

Between November 2013 and February 2021, six surgeons performed 174 RALPNs for clinical T1 kidney tumors in our institute. Of these, 114 RALPNs were performed by a single surgeon (T.H.) and were analyzed in this study. The clinical staging and the RENAL score were determined based on CT scans.

RALPN was performed using standard transperitoneal or retroperitoneal approaches depending on the patient’s history of trans-abdominal surgery, the tumor size, and the tumor location. We did not use a ureteral catheter [15]. Partial nephrectomy was performed by clamping the main trunk of a renal artery(s) with a bulldog clamp. In renal hilar tumors, a renal vein was also clamped. Tumors with a 5-mm surgical margin were resected under 15 mm Hg CO_2_ pressure, while the preparation, renal capsule, and tumor surface were exposed under 10 mm Hg CO_2_ pressure. The inner medullary hemostatic sutures were performed by a running barbed suture, followed by early unclamping and additional hemostatic sutures. Both the inner- and outer-layer renorrhaphy were performed by 2-0 STRATAFIX^TM^ Spiral PDS^TM^ Plus (Ethicon, USA) or 3-0 V-Loc^TM^ (Medtronic, Ireland), according to the tumor size and location. All specimens were removed using an ENDOPOUCH^TM^ RETRIEVER^TM^ Specimen bag (AutoSuture, UK) through a camera port. Intra- and postoperative complications were categorized based on the Satava [16] and the Clavien-Dindo [17] classification systems, respectively, into major or minor complications; major complications were defined as grade II or higher. To assess clinical risk factors for prolonged WIT, the patients were divided into three groups based on the number of RALPNs in chronological order (period 1: cases 1–38, period 2: cases 39–76, and period 3: cases 77–114).

### Ethics statement

All procedures were performed according to the tenets of the 1964 Declaration of Helsinki. This study consisted of a retrospective chart review. Informed consent was received for all the participating patients. Research involving human participants was supervised and approved by the Ethics Board of the Akita University Hospital.

### Statistics analysis

All data were expressed as the median (range). The clinical risk factors associated with a longer WIT were screened by univariate analysis. The factors with a *p*-value of < 0.1 in the univariate analysis were confirmed by logistic regression analysis. All *p*-values were two-sided, and *p* < 0.05 was considered statistically significant. All statistical analyses were performed using SPSS version 26.0 statistical software (SPSS Japan Inc., Tokyo, Japan).

### Data availability statement

The data presented in this study are available on request from the corresponding author.

## Results

Tables 1 and 2 present the clinicopathological characteristics of all enrolled patients. The tumor diameter increased with each period (median; 25.7 mm vs. 33.9 mm vs. 38.9 mm, *p* < 0.01), but there was no difference in WIT (17.2 min vs. 18.4 min vs. 17.4 min, *p* = 0.666). This is perhaps because the indications for tumor diameter and kidney function extended with increasing surgical experience. In period 1, only one patient (2.6%) had a tumor stage higher than Ib, but the incidence rate increased in period 2 (8 patients, 21.1%) and period 3 (18 patients, 47.4%) (*p* < 0.01 [chi-squared test]). There was no difference in the complication rate among periods (all periods: 6 incidences, 15.8%, *p* = 1.000). Risk factors for longer WIT were screened per period by dividing the groups by the median WIT (17.2 min; Supplementary Table 1). Logistic regression analysis confirmed that male patients (odds ratio [OR], 6.182; 95% confidence interval [CI], 1.101–34.700; *p* = 0.038) and a RENAL N-component score of 3 (OR, 6.500; 95% CI, 1.127–34.484; *p* = 0.036) were significantly associated with longer WIT in period 1 (Table 3). In period 2, tumors larger than 33 mm (OR, 6.500; 95% CI, 1.537–27.486; *p* = 0.011), a RENAL N-component score of 3 (OR, 5.000; 95% CI, 1.096–22.820; *p* = 0.038), and a total RENAL score of 8 or more (OR, 4.667; 95% CI, 1.187–18.352; *p* = 0.027) were significantly associated with longer WIT (Table 3). In period 3, a RENAL L-component score of 3 was significantly associated with longer WIT (OR, 3.900; 95% CI, 1.004–15.276; *p* = 0.044; Table 3), but the tumor diameter and the total RENAL score were not (Supplementary Table 1).

**Table 1:** Patient characteristics

		All cases	All cases by TH	1st period	2nd period	3rd period
		*N* = 174	*N* = 114	*N* = 38	*N* = 38	*N* = 38
Age, years (median [IQR])		64 (56–70)	64 (58–70)	62.4 (58–68)	65.5 (59–70)	64 (55–70)
Sex (%)	Male	129 (74.1)	85 (74.6)	28 (73.7)	32 (84.2)	25 (65.8)
	Female	45 (25.9)	29 (25.4)	10 (26.3)	6 (15.8)	13 (34.2)
BMI, kg/m^2^ (median [IQR])		24.5 (22.5–26.7)	24.4 (22.0–26.6)	24.2 (21.5–25.5)	24.6 (22.5–27.4)	24.6 (21.1–26.7)
Laterality (%)	Right	107 (61.5)	69 (60.5)	26 (68.4)	26 (68.4)	17 (44.7)
	Left	67 (38.5)	45 (39.5)	12 (31.6)	12 (31.6)	21 (55.3)
Diameter, mm (median [IQR])		29 (22–35)	32 (25–41)	26 (20–30)	33.5 (27–42)	39.5 (30–47)
RENAL nephrometry score (median [IQR])		7 (5–8)	7 (5–8)	5 (4–7)	7 (6–8)	8 (7–9)
R (median [IQR])		1 (1–1)	1 (1–1)	1 (1–1)	1 (1–1)	1 (1–2)
E (median [IQR])		2 (1–2)	2 (1–2)	1 (1–2)	2 (1–2)	2 (1–2)
N (median [IQR])		2 (1–3)	3 (1–3)	1 (1–3)	3 (2–3)	3 (3–3)
L (median [IQR])		1 (1–3)	1 (1–3)	1 (1–2)	2 (1–3)	2 (1–3)
Preoperative eGFR, mL/min (median [IQR])		67.6 (55.8–75.5)	69.4 (58.7– 76.4)	75.2 (65.2–78.0)	64.7 (55.7–75.0)	68.2 (54.1–75.8)
POM 1 eGFR, mL/min (median [IQR])		63.4 (52.5–75.9)	63.9 (53.9–77.5)	67.9 (62.2–82.2)	64.9 (52.8–7.3)	55.4 (46.7–67.6)
Clinical T (%)	1a	147 (84.5)	87 (76.3)	37 (97.4)	30 (78.9)	20 (52.6)
	1b	27 (15.5)	27 (23.7)	1 (2.6)	8 (21.1)	18 (47.4)

*IQR* interquartile range; *BMI* body mass index; *RENAL* radius, exophytic/endophytic, nearness of tumor to collecting system, anterior/posterior, location relative to polar line; *eGFR* estimated glomerular filtration rate; *POM* postoperative month; *T* size of the primary tumor and whether it has invaded nearby tissue; *TH* high-volume surgeon in our hospital

**Table 2:** Surgical outcomes of robot-assisted partial nephrectomy

		All cases	All cases by HT	1st period	2nd period	3rd period
Median (range)		*N* = 174	*N* = 114	*N* = 38	*N* = 38	*N* = 38
Approach (%)	Transperitoneal	114 (65.5)	69 (60.5)	31 (81.6)	27 (71.1)	11 (28.9)
	Retroperitoneal	60 (34.5)	45 (39.5)	7 (18.4)	11 (28.9)	27 (71.1)
Operative time (min)		221 (140–399)	223 (140–353)	197.5 (140–353)	231 (153–290)	239.5 (174–328)
Operator		6	1	1	1	1
Console time (min)	All cases	143 (42–277)	138.5 (42–277)	140.5 (64–203)	142 (74–200)	136.5 (42–277)
	Transperitoneal	157 (42–262)				
	Retroperitoneal	113 (58–277)				
Estimated blood loss (mL)		42 (0–1805)	60 (0–1805)	63 (0–1805)	50.5 (0–1184)	74.5 (0–736)
Warm ischemia time (min)		17.3 (9.2–56)	17.2 (9.2–56.0)	17.4 (9.3–34.0)	16.9 (10.1–56.0)	17.3 (10.9–29.2)
Intra-operative complication (%)	Satava ≥ 2	3 (1.7)	1 (0.9)	1 (2.6)	0 (0)	0 (0)
Postoperative complication (%)	Clavien-Dindo ≥ 2	27 (15.5)	17 (14.9)	5 (13.2)	6 (15.8)	6 (15.8)
Positive surgical margin (%)		1 (0.6)	1 (0.9)	1 (2.6)	0 (0)	0 (0)
eGFR at 1 month after operation (%)	≥ 90%	121 (69.5)	73 (64.0)	29 (76.3)	28 (73.7)	16 (42.1)
Trifecta accomplishment (%)		151 (86.8)	100 (87.7)	33 (86.8)	32 (84.2)	35 (92.1)
Histology (%)	Clear	128 (73.6)	84 (73.7)	27 (71.1)	33 (86.8)	24 (63.2)
	Papillary	22 (12.6)	14 (12.3)	4 (10.5)	3 (7.9)	7 (18.4)
	Chromophobe	9 (5.2)	7 (6.1)	2 (5.3)	2 (5.3)	3 (7.9)
	AML	7 (4.0)	5 (4.4)	3 (7.9)	0 (0)	2 (5.3)
	Oncocytoma	3 (1.7)	3 (2.6)	2 (5.3)	0 (0)	1 (2.6)
	Xp11.2	1 (0.6)	1 (0.9)	0 (0)	0 (0)	1 (2.6)
	MTSCC	1 (0.6)	0 (0)	0 (0)	0 (0)	0 (0)
Pathological T (%)	1a	145 (83.3)	90 (78.9)	31 (81.6)	33 (86.8)	26 (68.4)
	1b	15 (8.6)	14 (12.3)	1 (2.6)	5 (13.2)	8 (21.1)
	3a	2 (1.1)	2 (1.8)	1 (2.6)	0 (0)	1 (2.6)

*HT* high-volume surgeon in our hospital; *eGFR* estimated glomerular filtration rate; *AML* angiomyolipoma; *MTSCC* mucinous tubular and spindle cell carcinoma; *T* size of the primary tumor and whether it has invaded nearby tissue

**Table 3:** Risk factors longer WIT in each period

		Risk category	OR	95% CI		*p*
				Lower limit	Upper limit	
1st period	Gender	Male	6.182	1.101	34.700	0.038
	N	3	6.500	1.127	34.484	0.036
2nd period	Tumor diameter (mm)	33 ≤	6.500	1.537	27.486	0.011
	N	3	5.000	1.096	22.820	0.038
	Total score	8 ≤	4.667	1.187	18.352	0.027
3rd period	L	3	3.900	1.004	15.276	0.044
	Total score	9 ≤	2.444	0.654	9.130	0.184

*WIT* warm ischemia time; *OR* odds ratio; *CI* confidence interval; *N* nearness of the tumor to the collecting system or sinus; *L* location relative to the polar lines

## Discussion

As the surgeon gained RALPN experience, the procedure was able to be used in more complex cases, such as patients with larger tumors and worse kidney function. However, the complication rate did not change throughout the study period. In the introductory period, male sex and a high RENAL N-component score were associated with longer WIT. In the intermediate period, a large tumor and high N-component and total RENAL scores were associated with longer WIT, but in the late period, only a high RENAL L-component score was associated.

Over the past decade, robotics rapidly spread across all surgical fields, with tremendous innovation in urological surgeries [18]. Presently, robot techniques are routinely applied safely and effectively to urological operations. The success of RALPN may be primarily influenced by a surgeon’s experience and confidence. In this study, the indication for RALPN for RCC was influenced by tumor size during the early period [19]. However, as the surgeon’s experience with RALPN increased, the indication for RALPN expanded, eventually becoming the standard procedure for T1a and T1b RCC at our institution. Other studies have also reported similar data [20, 21]. In our early period, tumors < 4 cm in diameter were suitable for the robotic platform, but tumors > 4 cm in size were also treated with RALPN in the later periods. In period 3, 47% of tumors were stage cT1b (18/38; Table 1), and RALPN was performed by our institute’s most experienced surgeon. Furthermore, during the late period, patients with less kidney function, such as RCC in the solitary kidney, were also candidates for RALPN.

Objectively evaluating the difficulty of a partial nephrectomy has been attempted ever since the introduction of the laparoscopic partial nephrectomy. The RENAL score is a well-validated method for this purpose and is used to evaluate RALPN patients. However, these scores are primarily validated from laparoscopic partial nephrectomies and may be inadequate for assessing robotic procedure difficulties [10, 22]. Nevertheless, each RENAL score component focused on representative points regarding the kidney tumor dissection. The N-component indicates the nearness of the tumor to the collecting system or sinus. For a less experienced surgeon, an open collecting system and a large vessel injury would be troublesome [23], potentially resulting in longer WIT during the early experience period. The L-component indicates the tumor location relative to the polar lines. A high L-component score included hilar tumors in patients with a large tumor. In our late period, most cases were considered highly complex tumors because of their large diameter (median, 39.5 mm), high N factor (median 3 [interquartile range 3-3]), and high total RENAL score (median, 8). In these complicated cases, a central tumor lesion affected WIT because a major parenchymal defect across a central kidney lesion is difficult to cope with. To suture between surgical margins, a deep suture is needed to avoid parenchymal fracture and postoperative hemorrhage. However, this suture potentially causes injury to major vessels and the collecting system [24]. Thus, careful suturing is required, resulting in longer WIT [25, 26].

Dissection and suturing skills evolve with increasing experience, and obtaining skills other than laparoscopic partial nephrectomy is a relatively rapid process. Zeuschner et al. reviewed RALPN outcomes and suggested that 35 cases were the minimum required number to acquire adequate RALPN skills [27]. The complication rate did not change throughout our study, which could be explained by the fact that the cases were carefully selected to match the surgeon’s skill during each period. In addition, the early unclamping technique may contribute to our stable WIT result [26]. Early unclamping allowed us for only focusing on the precise dissection of the tumor because this technique can provide information regarding arterial bleeding from the resected bed before renorrhaphy. Early unclamping significantly reduces the risk of asymptomatic unruptured pseudoaneurysm [27].

A pseudoaneurysm was the main complication in this study. As RALPN use increased, fewer pseudoaneurysm cases after laparoscopic partial nephrectomy were expected [28]. However, pseudoaneurysms remain at a low but constant rate in the robotic era [29]. In this study, all pseudoaneurysm cases were successfully managed by arterial embolization. Based on these findings derived and our study’s complication rate, we conclude that RALPN is a safe procedure.

Our study had several limitations. First, this was a retrospective review of data from patients treated at a single institution, and multi-center, prospective studies are still needed. Second, WIT risk evaluation was only investigated by RENAL score-related factors and clinical factors and did not include C-index or PADUA score. Third, long-term follow-up of kidney function was lacking. Almost all of our WIT was less than 25 min, and it is uncertain if such a short WIT affects kidney function. Although this is not the first study on this topic, to our knowledge, ours is the first to assess WIT risk factors in complex patients. Furthermore, a single surgeon performed surgery on a sufficient number of patients, emphasizing the relevance of our study results.

## Conclusions

RALPN is an effective and safe treatment that combines the advantages of a robotic platform with the standard oncological surgical strategies. A large tumor in the central lesion of a kidney may result in longer WIT, even when the procedure is performed by an experienced surgeon.

## Supplementary Information

**Additional file 1: Supplement Table 1.** Risk factor screening of the patients divided into three periods in chronological order.

## Data Availability

Data could be accessed on request from the authors.

## References

[1] Ljungberg B, Bensalah K, Canfield S, Dabestani S, Hofmann F, Hora M, Kuczyk MA, Lam T, Marconi L, Merseburger AS (2015). EAU guidelines on renal cell carcinoma: 2014 update. Eur Urol.

[2] Andrade HS, Zargar H, Caputo PA, Akca O, Kara O, Ramirez D, Haber GP, Stein RJ, Kaouk JH (2016). Five-year oncologic outcomes after transperitoneal robotic partial nephrectomy for renal cell carcinoma. Eur Urol.

[3] Deng W, Li J, Liu X, Chen L, Liu W, Zhou X, Zhu J, Fu B, Wang G (2020). Robot-assisted versus laparoscopic partial nephrectomy for anatomically complex T1b renal tumors with a RENAL nephrometry score >/=7: a propensity score-based analysis. Cancer Med.

[4] Mikhail D, Sarcona J, Mekhail M, Richstone L (2020). Urologic robotic surgery. Surg Clin North Am.

[5] Hung AJ, Cai J, Simmons MN, Gill IS (2013). “Trifecta” in partial nephrectomy. J Urol.

[6] Volpe A, Blute ML, Ficarra V, Gill IS, Kutikov A, Porpiglia F, Rogers C, Touijer KA, Van Poppel H, Thompson RH (2015). Renal ischemia and function after partial nephrectomy: a collaborative review of the literature. Eur Urol.

[7] Chronic Kidney Disease Prognosis C, Matsushita K, van der Velde M, Astor BC, Woodward M, Levey AS, de Jong PE, Coresh J, Gansevoort RT. Association of estimated glomerular filtration rate and albuminuria with all-cause and cardiovascular mortality in general population cohorts: a collaborative meta-analysis. Lancet. 2010;375(9731):2073–81. 10.1016/S0140-6736(10)60674-5 PMC3993088 20483451

[8] Veccia A, Antonelli A, Uzzo RG, Novara G, Kutikov A, Ficarra V, Simeone C, Mirone V, Hampton LJ, Derweesh I (2020). Predictive value of nephrometry scores in nephron-sparing surgery: a systematic review and meta-analysis. Eur Urol Focus.

[9] Yao Y, Xu Y, Gu L, Liu K, Li P, Xuan Y, Gao Y, Zhang X (2020). The Mayo adhesive probability score predicts longer dissection time during laparoscopic partial nephrectomy. J Endourol.

[10] Kumar RM, Lavallee LT, Desantis D, Cnossen S, Mallick R, Cagiannos I, Morash C, Breau RH (2017). Are renal tumour scoring systems better than clinical judgement at predicting partial nephrectomy complexity?. Can Urol Assoc J.

[11] Okhunov Z, Rais-Bahrami S, George AK, Waingankar N, Duty B, Montag S, Rosen L, Sunday S, Vira MA, Kavoussi LR (2011). The comparison of three renal tumor scoring systems: C-Index, P.A.D.U.A., and R.E.N.A.L. nephrometry scores. J Endourol.

[12] Hu JC, Treat E, Filson CP, McLaren I, Xiong S, Stepanian S, Hafez KS, Weizer AZ, Porter J (2014). Technique and outcomes of robot-assisted retroperitoneoscopic partial nephrectomy: a multicenter study. Eur Urol.

[13] Imbeault A, Pouliot F, Finley DS, Shuch B, Dujardin T (2012). Prospective study comparing two techniques of renal clamping in laparoscopic partial nephrectomy: impact on perioperative parameters. J Endourol.

[14] Omidele OO, Davoudzadeh N, Palese M. Trifecta outcomes to assess learning curve of robotic partial nephrectomy. JSLS. 2018;22(1):e2017.00064. 10.4293/JSLS.2017.00064 PMC5809150 29472757

[15] Nishimura K, Sawada Y, Sugihara N, Funaki K, Koyama K, Noda T, Fukumoto T, Miura N, Miyauchi Y, Kikugawa T (2021). A low RENAL Nephrometry Score can avoid the need for the intraoperative insertion of a ureteral catheter in robot-assisted partial nephrectomy. World J Surg Oncol.

[16] Tepeler A, Resorlu B, Sahin T, Sarikaya S, Bayindir M, Oguz U, Armagan A, Unsal A (2014). Categorization of intraoperative ureteroscopy complications using modified Satava classification system. World J Urol.

[17] Clavien PA, Barkun J, de Oliveira ML, Vauthey JN, Dindo D, Schulick RD, de Santibanes E, Pekolj J, Slankamenac K, Bassi C (2009). The Clavien-Dindo classification of surgical complications: five-year experience. Ann Surg.

[18] Honda M, Morizane S, Hikita K, Takenaka A (2017). Current status of robotic surgery in urology. Asian J Endosc Surg.

[19] Motoyama D, Matsushita Y, Watanabe H, Tamura K, Suzuki T, Ito T, Sugiyama T, Otsuka A, Miyake H (2020). Initial learning curve for robot-assisted partial nephrectomy performed by a single experienced robotic surgeon. Asian J Endosc Surg.

[20] Vartolomei MD, Matei DV, Renne G, Tringali VM, Crisan N, Musi G, Mistretta FA, Russo A, Cozzi G, Cordima G (2019). Robot-assisted partial nephrectomy: 5-yr oncological outcomes at a single European tertiary cancer center. Eur Urol Focus.

[21] Tufek I, Mourmouris P, Doganca T, Obek C, Argun OB, Tuna MB, et al. Robot-assisted partial nephrectomy for T1b tumors: strict trifecta outcomes. JSLS. 2017;21(1):e2016.00113. 10.4293/JSLS.2016.00113 PMC5357684 28352149

[22] Rai BP, Patel A, Abroaf A, Suleyman N, Gowriemohan S, Prasad V, Vasdev N, Adshead J (2017). External validation of four nephrometry scores for trans-peritoneal robotic partial nephrectomy. Cent European J Urol.

[23] Mayer WA, Godoy G, Choi JM, Goh AC, Bian SX, Link RE (2012). Higher RENAL Nephrometry Score is predictive of longer warm ischemia time and collecting system entry during laparoscopic and robotic-assisted partial nephrectomy. Urology.

[24] Chavali JSS, Nelson R, Maurice MJ, Kara O, Mouracade P, Dagenais J, Reese J, Bayona P, Haber GP, Stein RJ (2018). Hilar Parenchymal Oversew: a novel technique for robotic partial nephrectomy hilar tumor renorrhaphy. Int Braz J Urol.

[25] Eyraud R, Long JA, Snow-Lisy D, Autorino R, Hillyer S, Klink J, Rizkala E, Stein RJ, Kaouk JH, Haber GP (2013). Robot-assisted partial nephrectomy for hilar tumors: perioperative outcomes. Urology.

[26] Yin X, Jiang S, Shao Z, Lu Y, Guo J, Xiao Y, Zhu X, Yu H, Ma H, Yang Y (2020). Kidney ventrally rotation technique in retroperitoneal robot-assisted partial nephrectomy for posterior hilar tumor: technical feasibility and preliminary results. World J Surg Oncol.

[27] Zeuschner P, Meyer I, Siemer S, Stoeckle M, Wagenpfeil G, Wagenpfeil S, Saar M, Janssen M (2021). Three different learning curves have an independent impact on perioperative outcomes after robotic partial nephrectomy: a comparative analysis. Ann Surg Oncol..

[28] Chung DY, Lee JS, Ahmad A, Chang KD, Ham WS, Han WK, Hong CH, Choi YD, Rha KH (2020). Lessons learned from clinical outcome and tumor features of patients underwent selective artery embolization due to postoperative bleeding following 2076 partial nephrectomies: propensity scoring matched study. World J Urol.

[29] Chavali JSS, Bertolo R, Kara O, Garisto J, Mouracade P, Nelson RJ, Dagenais J, Kaouk JH (2019). Renal arterial pseudoaneurysm after partial nephrectomy: literature review and single-center analysis of predictive factors and renal functional outcomes. J Laparoendosc Adv Surg Tech A.

