# Supplementary material for: Factors influencing warm ischemia time in robot-assisted partial nephrectomy change depending on the surgeon’s experience
Source: World J Surg Oncol. 2022 Jun 15;20:202. doi: 10.1186/s12957-022-02669-0 (PMC9199197; doi:10.1186/s12957-022-02669-0)
Supplement: Supplementary file 1 — Additional file 1: Supplement Table 1. Risk factor screening of the patients divided into three periods in chronological order. [file 12957_2022_2669_MOESM1_ESM.docx]

| Supplement Table 1 Risk factor screening of the patients divided into three periods in chronological order. | | | | | | | |  |
| --- | --- | --- | --- | --- | --- | --- | --- | --- |
|  |  | 1st period | | 2nd period | | 3rd period | |  |
|  |  | N = 38 | | N = 38 | | N = 38 | |  |
| WIT (min) | | < 17.2 vs 17.2 ≤ | | < 17.2 vs 17.2 ≤ | | < 17.2 vs 17.2 ≤ | |  |
| Continuous variable | |  |  |  |  |  |  |  |
|  |  | median | p | median | p | median | p |  |
|  | Age (year) | 65.0 vs 60.0 | 0.385 | 66.0 vs 64.5 | 0.762 | 63.0 vs 64.5 | 0.74 |  |
|  | BMI (kg/m2) | 23.3 vs 24.5 | 0.103 | 24.6 vs 23.3 | 0.433 | 25.5 vs 23.3 | 0.393 |  |
|  | Tumor diameter (mm) | 22.0 vs 28.0 | 0.123 | 29.0 vs 35.5 | 0.017 | 40.0 vs 39.5 | 0.460 |  |
| Nominal variable | |  |  |  |  |  |  |  |
|  |  | number | p | number | p | number | p |  |
|  | Gender (Male) | 11 vs 17 | 0.038 | 17 vs 15 | 1.000 | 12 vs 13 | 1.000 |  |
|  | Laterality (Right) | 14 vs 12 | 0.728 | 14 vs 12 | 1.000 | 8 vs 10 | 0.757 |  |
|  | Approach (retroperitoneal) | 2 vs 5 | 0.405 | 7 vs 4 | 0.485 | 13 vs 14 | 1.000 |  |
|  | R (1 <) | 0 vs 1 | 1.000 | 3 vs 5 | 0.438 | 8 vs 10 | 0.757 |  |
|  | E (1 <) | 8 vs 10 | 0.746 | 10 vs 12 | 0.342 | 11 vs 17 | 0.144 |  |
|  | N (2 <) | 2 vs 9 | 0.029 | 10 vs 15 | 0.043 | 13 vs 16 | 0.709 |  |
|  | L (1 <) | 5 vs 7 | 0.728 | 9 vs 12 | 0.185 | 5 vs 12 | 0.045 |  |
|  | Total score (7 <) | 1 vs 4 | 0.340 | 6 vs 12 | 0.050 | 9 vs 16 | 0.087 |  |
|  | Clinical T stage (T1b) | 0 vs 1 | 1.000 | 3 vs 5 | 0.438 | 8 vs 10 | 0.757 |  |
|  |  |  |  |  |  |  |  |  |
| WIT, warm ischemia time; BMI, body mass index; R, radius; E, exophytic/endophytic properties; N, nearness of the tumor to the collecting system or sinus; L, location relative to the polar lines; T, size of the primary tumor and whether it has invaded nearby tissue | | | | | | | |  |
|  |  |  |  |  |  |  |  |  |
|  |  |  |  |  |  |  |  |  |
